# Deaths of Female Sex Workers from HIV/AIDS in Kenya, Nigeria, and the Democratic Republic of the Congo 2019–2023 and Barriers to Antiretroviral Therapy Adherence

**DOI:** 10.3390/ijerph23030318

**Published:** 2026-03-04

**Authors:** Alexa Stefanko, Byron A. Foster, Brian Willis, Patrick Ezie, Emily Perttu, Wendy L. Macias-Konstantopoulos

**Affiliations:** 1 Global Health Promise, Portland, OR 97201, USA; stefanka@ohsu.edu (A.S.); fosterb@ohsu.edu (B.A.F.);; 2 School of Public Health, Oregon Health & Science University and Portland State University, Portland, OR 97239, USA; 3 Department of Pediatrics, Oregon Health & Science University, Portland, OR 97239, USA; 4 Silver Cross Hospital, Abuja 900211, Nigeria; 5 Center for Social Justice and Health Equity, Department of Emergency Medicine, Massachusetts General Hospital, Harvard Medical School, Boston, MA 02114, USA

**Keywords:** sex workers, HIV infection, antiretroviral therapy, food insecurity, sub-Saharan Africa, medication adherence

## Abstract

**Highlights:**

**Public health relevance—How does this work relate to a public health issue?**

- This study uses a community knowledge approach to identify 445 female sex workers (FSWs) who died from HIV/AIDS deaths across Kenya, Nigeria, and the DRC from 2019 to 2023.
- This study examines barriers to antiretroviral therapy (ART) adherence among FSWs and investigates relationships between death from HIV/AIDS and food insecurity, behavioral health, and stigma.

**Public health significance—Why is this work of significance to public health?**

- A total of 95.1% of FSWs who died of HIV/AIDS were not taking ART at the time of their death, and 89.5% had stopped taking ART rather than never initiating, indicating sustained adherence, rather than initial availability, as being the dominant gap.
- The leading barriers to non-adherence were food insecurity (22.9%), depression (13.5%), alcohol or drug use (10.4%), and stigma (5.7%).

**Public health implications—What are the key implications or messages for practitioners, policy makers and/or researchers in public health?**

- Programs seeking to reduce mortality from HIV/AIDS in FSWs should address food insecurity, mental health care, substance use treatment, and stigma reduction, as improving ART access alone is unlikely to reduce mortality.

**Abstract:**

Introduction: Female sex workers (FSWs) face heightened health risks, particularly from HIV, which accounts for 8% of FSW deaths in low- and middle-income countries. Adherence to antiretroviral therapy (ART) remains suboptimal and few studies have explored drivers of ART non-adherence among FSWs who died from HIV in sub-Saharan Africa. Methods: Using a community knowledge approach methodology, data on HIV-related deaths among FSWs were collected from a convenience sample across Kenya, Nigeria, and the Democratic Republic of the Congo between 1 February 2022, and 28 February 2023. Participants were recruited through local sex worker organizations and non-governmental organizations. A structured questionnaire was used to collect demographic data, HIV treatment history, and reasons for ART non-adherence from participants about the deaths of FSWs they knew of personally. A qualitative descriptive analysis was used to code the interviews for reasons for ART non-adherence. Results: A total of 853 FSWs reported the deaths of 445 peers who had died from HIV/AIDS. The mean age at death was 27 years; 70.6% were mothers. Among the deceased, 95.1% were not taking antiretrovirals at the time of their death, and 90% had stopped using ART, rather than never initiating treatment. The most cited reasons for ART non-adherence were food insecurity (97; 22.9%), alcohol and drug use (44; 10.4%), depression (57; 13.5%), and stigma (24; 5.7%). Conclusion: HIV-related deaths among FSWs in these three sub-Saharan African countries are associated with food insecurity, mental health issues, substance use, and stigma. Improving access to ART alone is insufficient to reduce mortality.

## 1. Introduction

Female sex workers (FSWs) face disproportionate health risks due to a combination of socioeconomic, behavioral, and systemic factors. A major contributor to mortality in this population is HIV infection, with the prevalence of HIV among FSWs in Eastern and Southern Africa reported as 36%, and HIV contributing to 7.4% of FSW deaths [1,2]. Despite global efforts to scale up HIV interventions, barriers such as stigma, social isolation, food insecurity, behavioral health challenges, and the criminalization of sex work contribute to suboptimal treatment outcomes [3]. Studies indicate that FSWs are 30 times more likely to be living with HIV compared to the general population, yet adherence to antiretroviral therapy (ART) remains low in many contexts. For example, in a 2016 cohort, only 43% of FSWs infected with HIV reported taking antiretrovirals [4,5]. ART adherence is critical for viral suppression and reduction in HIV transmission, with direct implications for population-level transmission and mortality

Systematic contributors to the lower use of ARVs in FSWs in other regions include high rates of food insecurity, which has been found to be associated with both lower adherence to ART, and more-rapid disease progression [6,7,8,9]. Beyond the adverse effects associated with taking ART without food, ART may be less effective amongst those who are experiencing malnutrition, leading to higher viral loads [10]. The COVID-19 pandemic further magnified existing vulnerabilities by restricting access to healthcare, exacerbating food insecurity, and contributing to conflict and social unrest [11].

Substance use and mental health challenges further complicate ART adherence among FSWs. Research shows that FSWs have disproportionately high rates of unhealthy substance use [12], which has been linked to reduced adherence to HIV treatment. Recent systematic reviews have found that 42% of FSWs in low- and middle-income countries (LMICs) reported recent substance use during sex work [13], and 41% report hazardous, harmful, or dependent alcohol use [14], both of which were correlated with lower ART adherence [15,16].

Mental health disorders, particularly depression, are highly prevalent yet underdiagnosed among FSWs [17] and they serve as significant barriers to ART adherence. A study in South Africa found that 80.9% of FSWs in KwaZulu-Natal had symptoms of depression, but only 15% were receiving psychiatric treatment [18]. FSWs experiencing depression are more likely to have an unsuppressed viral load, and individuals living with HIV and depressive symptoms are 42% less likely to achieve good adherence with ART [19,20]. While prior studies have examined HIV morbidity among FSWs and the paucity of ART adherence among FSWs in other regions, there is a lack of research specifically documenting the magnitude and intensity with which barriers such as food insecurity, substance use, and stigma converge among FSWs. Effective and targeted interventions to improve ART adherence and therefore reduce morbidity from HIV cannot be designed without insight into how these structural and behavioral barriers manifest in FSW populations.

Several factors contribute to this research gap. Weak vital statistics systems in LMICs result in limited mortality data in resource-poor and hard-to-reach populations such as FSWs, refugees, and displaced persons. The societal stigma surrounding sex work further compounds this issue, making it difficult to gather accurate mortality information. This lack of data presents a significant challenge in developing targeted interventions for FSW populations. In addition, HIV among FSWs is generally under surveillance to determine HIV rates, without a similar level of interest or funding to identify the causes of HIV mortality among FSWs.

The objective of this descriptive epidemiological study was to evaluate patterns and contributing factors to ART non-adherence. By interviewing FSWs with direct knowledge of the deaths of other FSWs from HIV, we collected valuable information about the social, behavioral, and systemic factors that contribute to fatal ART non-adherence. To our knowledge, this is the first study of this magnitude to systematically document the reasons for ART non-adherence that resulted in death of FSWs using a community knowledge approach (CKA) methodology, providing data critical to developing targeted prevention strategies.

## 2. Materials and Methods

### 2.1. Study Design and Participant Recruitment

This sub-analysis is part of a larger cross-sectional maternal–child health study conducted across three countries between 1 February 2022, and 28 February 2023, designed to investigate causes of death amongst FSWs and their children. Using the CKA methodology, local sex worker organizations (SWOs) and non-governmental organizations (NGOs) that provide services to FSWs recruited eligible FSWs to report deaths of FSWs in their community after 1 January 2019. These discussions took place in three cities in Kenya, three cities in Nigeria, and two cities in the Democratic Republic of the Congo (DRC) (Table 1). Country site selection was based on criteria including a large number of FSWs, a high HIV infection rate in FSWs, and the presence of local partner organizations. The cities and specific sites for convening the FSW groups were chosen based on the recommendations and expertise of local SWOs and NGOs, and where we conducted a similar study in 2019.

Criteria for participation included the following: age > 18 years; engaged in full-time sex work during the preceding 3 years; one living child age five or younger; and interaction with other sex workers in the community. The third criterion was included because mothers with young children are more likely to be socially engaged with other FSWs in their community through communal child-rearing and to understand the unique challenges of maternal–child health. Based on the inclusion criteria, local partners recruited eligible FSWs from local hotspots. Participants were informed that study participation was voluntary and would not impact the services they received.

### 2.2. Data Collection

In this study, we used a CKA methodology to investigate the factors contributing to HIV-related deaths among FSWs in Kenya, Nigeria, and the Democratic Republic of the Congo (DRC). In settings where weak vital statistics systems underestimate mortality, particularly among marginalized and criminalized populations such as FSWs, alternate epidemiological approaches are necessary [21]. The CKA is a low-cost, rapid, and validated approach for identifying under-documented deaths by leveraging social networks where health information is often shared. The CKA has been shown to have a high sensitivity in other low-resource settings; for example, it demonstrated a sensitivity of 80–100% for identifying neonatal deaths, stillbirths, jaundice-related deaths in ages ≥14 years, and maternal deaths [22,23,24].

After obtaining informed consent, a structured survey was administered to each participant to collect data on the deaths of other FSWs. For each death reported, participants were asked to provide demographic information of the deceased woman, including the date of death (day, month, year); location of death (city and country); age at time of death; if pregnant, gestational age; number of living children; and who is now caring for the living children. Details about the death were collected to determine the cause of death and the circumstances surrounding the death. For every death, regardless of the cause, participants were asked if the deceased FSW was infected with HIV, died as a result of HIV, was on ART at time of death and, if not, why not, and where the deceased woman died. Specifically for HIV-related deaths, participants were asked to provide information about ART adherence and reasons for non-adherence. ART adherence categorization was based on the report of the interviewee, not objective clinical or pharmacy records. This methodology is based on the close living relationship among many FSWs who often share with their friends and co-workers their health issues, including their HIV status, their ARV adherence status, and any reasons for non-adherence. During data collection, the lead researcher assigned a cause of death based on the information provided by each participant. Each report of a death from HIV/AIDS was reviewed by other members of the team (AS, EP) and any potentially miscoded causes of death were flagged for further review.

### 2.3. Data Management and Verification

Field data collection was overseen by the lead researcher, BW, who took detailed notes, including verbatim statements on the deaths reported. One staff member of the local partner organization was trained to assist in data collection. Interviews were conducted in English in Kenya and Nigeria. In the DRC, the staff member of the partner organization was trained in the use of the questionnaire by the lead researcher (BW), who administered the questions while the local staff translated questions and responses. Potential duplicate reports were identified by both the lead researcher and local staff assisting in data collection. If it was determined that a death had already been reported based on the name, age, location of death, and cause of death, the information on the duplicate report was not recorded. At the completion of data collection, BW and local staff reviewed the reports to identify any potential duplicate reports. Causes of death were initially coded by BW during data collection based on participant input and then reviewed and agreed upon by other members of the research team. The dataset was then subdivided by the cause of death.

The subset of responses from survey participants who died from HIV infection was collated in an Excel Version 2503 spreadsheet (Microsoft Corporation, Redmond, WA, United States). For each FSW who was not taking ARVs at the time of their death, a reason for non-adherence was assigned. If two reasons were provided and given equal weight, both were recorded. Additional contextual factors described in the narratives, such as COVID-19 disruptions, were recorded as ancillary themes and informed the qualitative insights presented in the results. A single coder (AS) initially categorized all reasons for non-adherence. Unclear or potentially miscoded causes of death or reasons for non-adherence were reviewed with BW. A consensus meeting between AS, BW, AF, and WLM led to the reclassification of 10 rationales for non-adherence. There was no need for external tiebreakers on rationale classification. Throughout the data collection and entry process, BW and another team member conducted regular quality checks to ensure accuracy and completeness.

### 2.4. Data Analysis

After the manual coding of ARV non-adherence rationales, the resulting dataset was analyzed using Python 3.9.6 (Python Software Foundation, www.python.org, accessed 10 April 2024). Totals for each rationale were calculated, and descriptive statistics were generated. As the goal of this analysis was descriptive in nature, statistical tests of comparison were not completed.

### 2.5. Research Ethics

All participants gave their written informed consent, which was formally recorded and witnessed. The study protocol was approved by the Institutional Review Board of Oregon Health & Sciences University (IRB ID: STUDY0002296) and the Ethics Review Committees in each of the three countries: Kenyata University Centre for Research Ethics and Safety in Kenya; Ecole De Sante Public, University de Kenshasha, DRC; and the Nigerian Institute of Medical Research, Institutional Review Board, Nigeria. Our ethical guidelines, standards, and disclosures meet or exceed those required under the Declaration of Helsinki.

### 2.6. Role of the Funding Source

The study sponsor was not involved in the study design, data collection, data interpretation, or the writing of this report, nor the decision to submit this paper for publication.

## 3. Results

Data were collected from 853 FSWs (Table 1) who reported 3810 deceased FSWs; of these deaths, 445 (11.7%) died from HIV/AIDS. The youngest reported death from HIV/AIDS was 11 years old (in the DRC in 2022), and the oldest reported death was 50 years old (in Nigeria in 2021). The mean age at death was 27.1 years, and 70.2% (n = 312) of the women had at least one living child. Twenty-eight (6.3%) of those who died were sexually exploited girls (aged ≤ 17) at the time of their death. Of these, three were reported to have been born with HIV, and the time and manner of infection were unclear for the remaining cases. Table 2 shows the distribution of deceased FSW reports per country and highlights the chronological distribution of reported HIV/AIDS-related deaths.

Overall, 95.1% of the girls and women who died of HIV/AIDS were reported to not be taking antiretroviral medications (ARVs) at the time of death, a pattern that remained largely consistent across the countries studied (Table 3). The majority of FSWs stopped taking ARVs (n = 379; 89.5%) rather than never initiating treatment (n = 32; 7.3%), with unclear information for the remaining cases (n = 12; 2.7%).

There was a clear documented reason for non-adherence in the case of 74.4% of FSWs who were not taking ARVs at the time of their death (Table 4). The most common reason in Kenya and Nigeria, cited in 30% of FSW deaths in each country, was difficulty acquiring food to take with ARVs. Overall, food insecurity was the single most cited barrier (n = 97, 22.9%) in this study. Additionally, the COVID-19 pandemic was reported to have exacerbated food insecurity for 13 Kenyan FSWs who died of HIV/AIDS. By contrast, food insecurity as the primary reason for ARV non-adherence was reported in only 7.6% of Congolese FSW deaths. In the DRC, depression (17.6%) and a lack of funds (10.7%) were the two most often reported reasons for non-adherence. Other frequently cited reasons for non-adherence included alcohol and drug use (n = 44, 10.4%), depression (n = 57, 13.5%), the stigma associated with an HIV diagnosis and sex work (n = 24, 5.7%), and denial of HIV or the effectiveness of the medication (n = 17, 4.0%).

The qualitative data from interviews provided further insights into barriers to ART adherence. Key themes included food insecurity, stigma, a fear of disclosing HIV status to partners or clients, a lack of financial resources, and mental health challenges. Participants frequently mentioned the difficulty of taking ART without adequate food, resulting in stomach pain and the eventual discontinuation of treatment. Thirteen participants noted that the COVID-19 pandemic exacerbated existing food insecurity and was the precipitating factor for ceasing regular ART treatment. Stigma and a fear of disclosure also played significant roles in non-adherence, as some FSWs stopped taking ART to prevent their partners or clients from discovering their HIV status. Depression and HIV diagnosis denial were particularly evident in participant quotes, with several expressing their colleagues’ sense of hopelessness about their treatment.

## 4. Discussion

This is one of the first studies to document the barriers to ART adherence for deceased FSWs who died of HIV/AIDS. This study identified that most (95.1%) of the FSWs who died of HIV were not taking ART at the time of their death. Only a small minority (7.3%) of the women who died of HIV/AIDS had never initiated therapy, highlighting that ART access barriers may be a minor factor in non-adherence, while a lack of basic needs, especially food, and depression were reported to be more significant factors in ART non-adherence.

Our findings align with those of prior studies from South Africa and Uganda that identify food insecurity, substance use, and occupational instability as barriers to ART adherence among FSWs living with HIV [25,26]. This prior work emphasized a fear of income loss, and work-related migration as mechanisms that interrupt treatment. Our results are in accordance, in that adherence challenges are rooted in structural, behavioral, and mental health factors rather than solely medication access. Our work extends the extant literature by demonstrating that these barriers were prevalent not just among FSWs living with HIV/AIDS, but also FSWs who died from HIV/AIDS.

Food insecurity has previously been described as a contributor to both engaging in sex work and reducing adherence to ART among women living with HIV [27]. Prior reports have described both nausea with taking ARTs without food and also misperceptions about the need to take food with ART, and food insecurity has been associated with poorer adherence to ART [28,29]. A recent randomized trial in HIV-infected adults (non-FSWs) showed that the provision of healthy foods improves adherence and mental health outcomes [30]. The COVID-19 pandemic and associated lockdown were mentioned as an exacerbating factor for food insecurity in 13 cases, all in Kenya. Prior qualitative studies have highlighted the effect of the pandemic on food insecurity and poverty among the urban poor in Kenya, emphasizing the disproportionate burden that restrictive measures may place on more disadvantaged individuals [31]. In Nigeria, food insecurity post-pandemic has persisted due to inflation [32]. The pandemic and associated restrictions had negative impacts on FSWs economically and through challenges accessing healthcare [33,34], which may have influenced the deaths here, given that this study spanned the pandemic period.

Substance use and mental health needs were described as being two contributors to non-adherence and the subsequent death of FSWs in our study. Substance use disorder among sex workers has been estimated at 35%, though with substantial international variation [35], and the prevalence of heavy episodic alcohol consumption has been estimated at between 47 and 56%, depending on the study [36]. Prior qualitative work revealed that FSWs’ use of alcohol or drugs both impairs their ability to remember to take ART and that they do not take ART with substances due to the concern for side effects [37]. Mental health concerns were associated with ARV nonadherence in 13.5% of FSWs across all countries and as high as 17.6% in the DRC. These observations align with those of prior studies, that found an association between depression and ART non-adherence [38]. Additional factors, such as HIV-related stigma, denial of diagnosis, and a fear of disclosure to clients or partners, also emerged as common themes. While these reasons were not always explicitly linked to depression or mental health, they likely interact with and exacerbate underlying mental health challenges in ways that our survey did not capture. We did not identify a role for religious leaders or herbal medications in association with adherence to ART, and those relationships are complex, with both positive and negative implications in the extant literature [39,40].

About 6.6% of the deaths from HIV occurred among sexually exploited girls under 18 years of age, and children are more susceptible than adults to sexual exploitation, HIV transmission, and drug and alcohol use disorders [41,42]. The proportion of girls under 18 who died from HIV varied significantly by country: 15.9% in the DRC, 4.6% in Nigeria, and 0% in Kenya. These differences underscore the need for country-specific interventions tailored to the unique profiles and vulnerabilities of each FSW population.

The recent defunding of HIV programs specifically serving FSWs in sub-Saharan Africa since early 2025 may exacerbate the vulnerabilities identified in this study. As our data illustrate, the deaths of FSWs from HIV/AIDS have effects that extend beyond the individual, leaving children at an increased risk of mother-to-child transmission of HIV, orphanhood, further physical and sexual exploitation, and death from malnutrition and other preventable causes in the absence of a mother’s care and protection [43,44,45]. The current study demonstrates that despite a pre-existing understanding of the increased barriers to ART adherence among FSWs more than a decade ago [3], these barriers persist and contribute to HIV mortality. Without sustained investment in community-led, integrated models of care that address the specific barriers experienced by FSWs (e.g., food insecurity, substance use, mental illness), preventable deaths among both women and children are likely to rise. Recent modeling data have also shown that programs that target not only ART availability, but also adherence, testing, and PrEP use for FSWs are not only effective, but cost-efficient [46]. Re-investment in programs that more comprehensively support FSWs living with HIV is essential and has the potential to improve treatment adherence, reduce preventable deaths, and protect the well-being of both women and their children.

### Limitations

This study has several limitations. First, data were collected through retrospective reports from fellow FSWs, which introduces the potential for recall bias. Second, the reporting FSWs may have been subject to social desirability bias, leading to the underreporting of stigmatized behaviors such as substance use or non-adherence to ART amongst their colleagues. Third, the use of a non-random convenience sample limits the generalizability of findings to the broader population of FSWs in these countries and precludes our ability to conduct statistically sound comparisons between countries. In particular, as we did not include childless FSWs, and our sample skewed towards younger participants, we are unable to generalize our findings to older or childless FSWs. Although participants reported the HIV-related deaths of FSWs as old as 50 years, we cannot determine if the relatively young mean ages at the time of death reflect a selection bias effect or shortened survival times of HIV infection in this population. Fourth, data were not collected from the FSWs at regular and routine intervals; rather, FSWs reported on the deaths of fellow FSWs in the preceding several years, which may introduce recency bias into the results. Nonetheless, the methodology’s focus on recent deaths is also a strength for public health surveillance. We aim to understand why FSWs are dying now, not five or ten years ago. The CKA captures the recent causes of death and related factors, such as food insecurity. Because FSWs often share health information within their social networks, deaths are more reliably reported by peers than by estranged family. In contrast to other methodologies that rely on family interviews, the CKA approach is better suited to populations whose families may be unaware of their work, unwilling to discuss it, or geographically distant and lacking relevant details. Lastly, the specific cause of death from HIV (e.g., which opportunistic infection) is not able to be determined with this method, and we lack access to the specific amount of time that FSWs were not taking their ART in terms of their progression of HIV/AIDS.

## 5. Conclusions

This study highlights that most FSWs described in our study population who died of HIV in Kenya, Nigeria, and the DRC had initiated and later discontinued ART due to food insecurity, substance use, untreated mental health conditions, and the intersection of HIV and occupational stigma. Prior work has called for HIV services to be integrated with efforts to address the unique needs of both FSWs and their children [44]. Our findings reinforce that ART availability alone is insufficient to prevent mortality from HIV/AIDS, and that interventions must also target the specific structural and behavioral factors that impede ART adherence in FSW populations in LMICs.

Without parallel efforts to improve food security, expand mental health services, and reduce stigma through community-led approaches, ART treatment adherence will remain low and mortality high. Future research efforts are needed to understand how addressing food insecurity and mental and behavioral health problems may improve ART adherence in these populations. Finally, given that 6% of HIV deaths involved sexually exploited girls under 18 years of age, this study serves as an urgent call for the study and implementation of targeted evidence-based policies and programs to prevent, protect, and preserve the health and lives of these young victims.

## Figures and Tables

**Table 1:** Date and locations of data collection; number of participants by city.

Country	City	Date of Data Collection	No. Participants	Total by Country
Kenya	Nairobi	10 February 2022–18 February 2022	103	302
	Mombasa	22 February 2022–27 March 2022	99	
	Kisumu	2 March 2022–8 March 2022	100	
Nigeria	Lagos	22 March 2022–31 March 2022	100	301
	Calabar	2 April 2022–14 April 2022	101	
	Abuja	20 April 2022–28 April 2022	100	
DRC	Bukavu	27 January 2023–5 February 2023	100	250
	Kinshasa	13 February 2023–28 February 2023	150	
			Total	853

**Table 2:** Data on female sex workers who died by year and country of residence *.

	Kenya (N = 177)	DRC (N = 138)	Nigeria (N = 130)	Overall (N = 445)
Age at death, mean (SD)	30 (6)	23 (7)	27 (8)	27 (7)
Age < 18 at death	0 (0%)	22 (15.9%)	6 (4.6%)	28 (6.3%)
Year of death				
2019	41	1	10	52
2020	52	--	16	68
2021	56	--	46	102
2022	28	70	58	156
2023	--	67	--	67

* Note that blank rows indicate no deaths reported for that year.

**Table 3:** ARV use status at the time of death.

	Kenya (N = 177)	DRC (N = 138)	Nigeria (N = 130)	Total (N = 445)
Taking ARVs, n (%)	7 (3.9%)	7 (5.0%)	8 (6.2%)	22 (4.9%)
Not taking ARVs, n (%)	170 (96.1%)	131 (95.0%)	122 (93.8%)	423 (95.1%)

**Table 4:** * Reasons for non-adherence in FSWs not taking ARVs at the time of HIV-related death.

	Kenya (N = 170)	DRC (N = 131)	Nigeria (N = 122)	Total (N = 423)
Difficulty acquiring food	51 (30.0%)	10 (7.6%)	36 (29.5%)	97 (22.9%)
Fear of client or partner finding out about HIV status	8 (4.7%)	1 (0.8%)	2 (1.6%)	11 (2.6%)
Stigma	11 (6.5%)	6 (4.6%)	7 (5.7%)	24 (5.7%)
Lack of funds to access ARVs	3 (1.8%)	14 (10.7%)	6 (4.9%)	23 (5.5%)
Denial of diagnosis or medication effectiveness	7 (4.1%)	3 (2.3%)	7 (5.7%)	17 (4.0%)
No time to get ARVs	0 (0%)	8 (6.1%)	0 (0%)	8 (1.9%)
Depression	24 (14.1%)	23 (17.6%)	10 (8.2%)	57 (13.5%)
Alcohol or drug use	31 (18.2%)	8 (6.1%)	5 (4.1%)	44 (10.4%)
Other	17 (10.0%)	25 (19.1%)	16 (13.1%)	58 (13.7%)
Unsure	18 (10.5%)	33 (25.2%)	33 (27.0%)	84 (19.8%)

* Percentages reflect the percentage of subjects who reported a factor as a primary reason for non-adherence. Some values sum to more than the total, as a small number of participants reported more than one reason for non-adherence.

## Data Availability

De-identified aggregate data used for this analysis can be requested from the Director of Global Health Promise after publication at bwillis@globalhealthpromise.org. No other documents will be made available. Access permission will be considered based on the following criteria: (a) the request should be for the purpose of partnering on research to address the needs of female sex workers or their children; or (b) for inclusion in a curriculum for educational purposes; or (c) for providing services to female sex workers and their children by governmental organizations, sex worker organizations, and non-government organizations. A request for one of these purposes will be considered from governmental organizations, sex worker organizations providing support to female sex workers or their children, or researchers from recognized institutions.
